# Machine Learning Applications in Population and Public Health: Guidelines for Development, Testing, and Implementation

**DOI:** 10.2196/68952

**Published:** 2025-10-24

**Authors:** Andrew D Pinto, Sharon Birdi, Steve Durant, Roxana Rabet, Rahul Parekh, Shehzad Ali, David Buckeridge, Marzyeh Ghassemi, Jennifer Gibson, Ava John-Baptiste, Jillian Macklin, Melissa D McCradden, Kwame McKenzie, Parisa Naraei, Akwasi Owusu-Bempah, Laura C Rosella, James Shaw, Ross Upshur, Sharmistha Mishra

**Affiliations:** 1 Upstream Lab, MAP Centre for Urban Health Solutions, Li Ka Shing Knowledge Institute, Unity Health Toronto, 30 Bond Street, Toronto, ON, M5B 1W8, Canada, 1 4168646060 ext 76148; 2 Department of Family and Community Medicine, St. Michael’s Hospital, Toronto, ON, Canada; 3 Department of Family and Community Medicine, Temerty Faculty of Medicine, University of Toronto, Toronto, ON, Canada; 4 Division of Clinical Public Health, Dalla Lana School of Public Health, University of Toronto, Toronto, ON, Canada; 5 Department of Family Medicine, College of Medicine, University of Saskatchewan, Saskatoon, SK, Canada; 6 Department of Epidemiology and Biostatistics, Western Centre for Public Health & Family Medicine, Western University, London, ON, Canada; 7 WHO Collaborating Centre for Knowledge Translation and Health Technology Assessment in Health Equity, Ottawa, ON, Canada; 8 Department of Health Sciences, University of York, York, United Kingdom; 9 Department of Epidemiology, Biostatistics and Occupational Health, School of Population and Global Health, McGill University, Montreal, QC, Canada; 10 Department of Electrical Engineering and Computer Science (EECS) and Institute for Medical Engineering and Science (IMES), Massachusetts Institute of Technology, Cambridge, MA, United States; 11 Joint Centre for Bioethics, University of Toronto, Toronto, ON, Canada; 12 Departments of Epidemiology and Biostatistics, Anesthesia and Perioperative Medicine, and Schulich Interfaculty Program in Public Health, Western University, London, ON, Canada; 13 Undergraduate Medical Education, Temerty Faculty of Medicine, University of Toronto, Toronto, ON, Canada; 14 Department of Bioethics, Hospital for Sick Children, Toronto, ON, Canada; 15 Australian Institute for Machine Learning, University of Adelaide, Adelaide, Australia; 16 Wellesley Institute, Toronto, ON, Canada; 17 Centre for Addiction and Mental Health, Toronto, ON, Canada; 18 Department of Computer Science, Toronto Metropolitan University, Toronto, ON, Canada; 19 Department of Sociology, Faculty of Arts and Sciences, University of Toronto, Toronto, ON, Canada; 20 Institute for Better Health, Trillium Health Partners, Toronto, ON, Canada; 21 Department of Laboratory Medicine and Pathobiology, Temerty Faculty of Medicine, Toronto, ON, Canada; 22 Division of Epidemiology, Dalla Lana School of Public Health, University of Toronto, Toronto, ON, Canada; 23 Department of Physical Therapy, Temerty Faculty of Medicine, University of Toronto, Toronto, ON, Canada; 24 Division of Infectious Diseases, Temerty Faculty of Medicine, University of Toronto, Toronto, ON, Canada; 25 MAP Centre for Urban Health Solutions, Li Ka Shing Knowledge Institute, Unity Health Toronto, Toronto, ON, Canada; 26 Management and Evaluation, and Division of Epidemiology, Institute of Health Policy, Dalla Lana School of Public Health, University of Toronto, Toronto, ON, Canada; 27 Institute of Medical Science, Faculty of Medicine, University of Toronto, Toronto, ON, Canada; 28 Institute for Clinical Evaluative Sciences, Toronto, ON, Canada

**Keywords:** population health, public health, machine learning, artificial intelligence, guideline, algorithmic bias, AI, health equity

## Abstract

Machine learning (ML), a subset of artificial intelligence, uses large datasets to identify patterns between potential predictors and outcomes. ML involves iterative learning from data and is increasingly used in population and public health. Examples include early warning of infectious disease outbreaks, predicting the future burden of noncommunicable diseases, and assessing public health interventions. However, ML can inadvertently produce biased outputs related to the quality and quantity of data, who is engaged and helping direct the analysis, and how findings are interpreted. Specific guidelines for using ML in population and public health have not yet been created. We assembled a diverse team of experts in computer science, statistical modeling, clinical and population health epidemiology, health economics, ethics, sociology, and public health. Drawing on literature reviews and a modified Delphi process, we identified five key recommendations: (1) prioritize partnerships and interventions to support communities considered structurally disadvantaged; (2) use ML for dynamic situations, such as public health emergencies, while adhering to ethical standards; (3) conduct risk assessments and bias mitigation strategies aligned with identified risks; (4) ensure technical transparency and reproducibility by publicly sharing data sources and methodologies; and (5) foster multidisciplinary dialogue to discuss the potential harms of ML-related bias and raise awareness among the public and public health community. The proposed guidelines provide operational steps for stakeholders, ensuring that ML tools are not only effective but also ethically grounded and feasible in real-world scenarios.

## Introduction

Machine learning (ML) is a form of artificial intelligence (AI) that is now used for a range of problems across many fields. ML involves a machine “learning” as it processes more data, improving predictive performance over time [1]. ML, as a set of tools, can be used for prediction, clustering, and causal inference. While prediction models are commonly used in public health to examine associations between potential predictors and outcomes, ML can also be used to identify groups with shared characteristics (clustering) and to identify potential causal associations between interventions and health outcomes [2,3]. However, these methods are often limited by the quality and quantity of the available data in public health [4].

As methods advance and as the availability of data increases, ML-based innovations are playing a central role in population and public health [5]. Key areas include the surveillance of infectious diseases [6]; predicting the burden of noncommunicable diseases (NCDs) [7]; and assessing public health interventions, including those focused on modifiable risk factors [8]. Awareness that ML could help improve population health is tempered by a growing understanding that it has risks and potentially negative consequences [9]. An important concern for the application of ML models is the perpetuation and amplification of biases reflecting patterns of societal inequality, when the accuracy of model predictions differs systematically across subpopulations, potentially leading to decisions that exacerbate health inequities [10]. This issue is rooted in differences in the amount and quality of data from different populations [11].

Bias in ML as it is applied to population and public health problems has been identified previously [12]; however, guidelines for ML applied to population and public health are limited. Without such guidelines, ML could exacerbate inequities and fail to adhere to methodological standards. For example, during the COVID-19 pandemic, ML models were used to predict infection rates and allocate health care resources. These models were found to be biased, particularly when data from underrepresented communities were either scarce or of lower quality, impacting the accuracy of predictions [13]. Public health organizations may lack the resources and expertise to thoroughly evaluate the ML models they use. While ethical frameworks for AI in health have emerged, they primarily focus on clinical settings. Public health involves unique applications, including population-level interventions, real-time surveillance, and communicable disease control, which require field-specific guidance. Our guidelines aim to address this gap by focusing on the distinct operational, ethical, and equity considerations involved in applying ML to public health. We aim to provide recommendations to those creating or revising ML tools for population and public health purposes, offer guidance to users of ML tools, and outline approaches on how to address bias. We centered our recommendations on the following questions: (1) What are best practices to identify and mitigate biases for those developing, testing, and implementing ML models in population health? (2) What are the priority areas for further research?

We used the GRADE (Grading of Recommendations Assessment, Development and Evaluation) [14], the National Institute for Health and Care Excellence (NICE) [15] approaches to guideline development, and other guidelines specific to epidemiology and health economics modeling [16,17]. We also drew insights from participatory frameworks for policy development governing ML [18] and from documentation on the governance of community data trusts, which provide data for many of the ML models we studied [19]. In addition, we considered international guidelines on AI ethics, such as the Montreal Declaration for Responsible Development of AI [20] and recommendations from the European Commission High-Level Expert Group on AI [21], along with the World Health Organization guidance on *Ethics & Governance of Artificial Intelligence for Health* [22].

## Guideline Panel Composition and Management of Competing Interests

To identify experts for the Delphi process, we consulted with leading researchers and practitioners in the fields of ML, public health, and ethics. We also searched online databases and professional networks to identify potential experts. We aimed to include experts from diverse backgrounds, including academia, industry, and government, with 17 chosen to be a part of the guideline panel (Multimedia Appendix 1). Our guideline panel includes academics in computer science, statistical modeling, epidemiology (clinical epidemiology, social epidemiology, infectious disease, and population health), health economics, ethics, sociology, primary care, public health, and the social determinants of health. Each member has contributed their disciplinary expertise and perspective regarding the social constructions influencing data, bias, and bias mitigation (Multimedia Appendix 1). Our panel members also bring diverse experiential knowledge, aligning with our guidelines’ emphasis on recognizing the importance of lived experience in equitable policy development. Panel members did not report any direct conflicts of interest. To ensure transparency, members also agreed to disclose any less direct or potential competing interests, but no such conflicts were found.

## Review of the Literature

We conducted a series of literature searches to retrieve existing guidelines related to population and public health, focusing on their intersections with equity, technical aspects, and knowledge mobilization. We searched a range of sources, including original research, reviews, commentaries, editorials, and gray literature (eg, government reports, policy documents, and organizational websites). In the course of this work, we drew on common elements found in other guidelines addressing bias in ML models [10,18,23,24]. These included practices such as engaging with communities impacted by biased models, prioritizing diversity in the teams developing and implementing models, considering which groups considered marginalized could benefit most from involvement in population and public health ML model development, ensuring robustness in the identification of groups considered vulnerable in data, systematically evaluating and reporting on bias, and conducting post-implementation evaluations to continue mitigating bias. In parallel, we conducted and reported three scoping reviews on ML applications in population and public health, specifically examining their use in addressing risk factors, NCDs, and communicable diseases. Two of these reviews have been published, providing an empirical foundation for our guidelines[25,26]. The first review found that bias mitigation was rarely addressed in ML studies focused on population health, particularly in the context of NCDs, with most efforts limited to addressing sex-related bias [25]. The second review, which examined ML applications for addressing risk factors for NCDs, found that although nine of the 20 studies mentioned algorithmic bias, these discussions were generally superficial and limited to traditional biases such as recall or misclassification [26]. To date, few systematic reviews have examined bias in ML applications for public health. Our findings are supported by another systematic review on the use of AI and ML in disaster response and public health emergencies, which similarly noted a lack of bias mitigation strategies despite the growing use of ML in these settings [27]. Our guidelines address this gap by offering operational recommendations that emphasize transparency, stakeholder engagement, and context-specific risk assessment [25]. Finally, we shared our evidence synthesis with the guideline committee alongside our draft recommendations.

## Topic Selection and Development of Recommendations

We used a modified Delphi approach to develop our recommendations, consisting of two iterative rounds. An initial list of relevant topics for ML applications in population and public health was compiled by SD and SB, based on a review of gray literature, academic publications, current practice reports, and consultations with the study team.

In round 1, this list was refined during three virtual workshops held on November 24, 2022, December 9, 2022, and December 13, 2022. These sessions focused on ethics and equity, technical considerations, and knowledge mobilization. During the workshops, participants assessed draft recommendations using a modified Likert scale and provided written feedback. SB and SD analyzed the quantitative scores and qualitative input to identify consensus and areas of divergence.

In round 2, we circulated the revised draft recommendations to the panel in advance of a follow-up virtual meeting. Panel members discussed the content and submitted additional written feedback. We incorporated these inputs to finalize the recommendations, drawing on frameworks such as GRADE and other relevant guideline development methodologies.

The final recommendations were organized into five thematic categories: (1) equity, diversity, and inclusion; (2) public health emergencies, deidentified population data use, and consent; (3) due diligence during model conceptualization and early development; (4) technical transparency, consistency, and data management; and (5) knowledge mobilization to the public, ML experts, and public health professionals.

## Recommendations

### Recommendation 1: Prioritize Partnerships and Interventions That Support Communities That Are Disadvantaged by Social and Economic Policies

This includes activities such as algorithmic bias mitigation, capacity building, and fostering equitable representation. Partnerships should not only engage diverse collaborators with expertise in ethical AI and public health but also invest in building local capacity to support sustainable and responsible ML deployment in diverse settings.

ML models may be prone to various types of bias, which can significantly impact the health and well-being of communities made vulnerable by social and economic policies. ML approaches that rely on data from internet searches or social media may lack representativeness, tending to exhibit a bias toward processing data from individuals with higher socioeconomic status, from certain age groups, or those living in urban areas rather than remote or rural areas [6]. Similarly, ML models used in population health often encounter missing or nonrepresentative data when relying on electronic medical records or public health data, which may not fully capture the diversity of the communities they aim to serve [28,29].

Members of diverse community groups, including communities disadvantaged by social and economic policies, bring a variety of perspectives and lived experiences to the table. These perspectives and insights must be actively prioritized in decision-making to identify and mitigate algorithm-related harms effectively [30]. Such experiences are crucial for meaningful research and transformative change within institutions. Expert consultation, while valuable, should not replace the direct involvement of people with lived experience; instead, it should complement it [31]. Unfortunately, those who stand to benefit most from fair and responsible ML applications are also those most at risk of algorithmic harm due to systemic biases and exclusion [32].

Prioritizing equity in ML requires balancing ethical considerations with technical feasibility and real-world implementation challenges. While transparency is critical for trust and accountability, full transparency can be resource intensive and difficult to achieve in low- and middle-income countries (LMICs), where data infrastructure and regulatory oversight may be limited. Similarly, while equity-driven approaches aim to reduce algorithmic harms, they require sustained investment in local expertise, which may not always be immediately available. Feasibility must therefore be considered at each stage, ensuring that solutions are both ethically sound and realistically implementable in diverse public health settings.

In LMICs, where these guidelines are particularly relevant, implementing ML partnerships and equity-focused interventions is often constrained by financial limitations, workforce shortages, and fragmented health data systems. Ensuring that ML applications promote equity while remaining feasible requires balancing ethical considerations with practical constraints. Many public health institutions in LMICs operate with competing priorities, making it challenging to allocate resources solely toward algorithmic transparency, bias mitigation, or extensive auditing processes. Policymakers, health care organizations, and ML developers in these settings must adopt scalable, context-aware approaches that allow for phased implementation. Resource-limited settings can start with standard approaches to data definition and extraction, streamlined documentation, internal monitoring, and bias assessments targeted at high-risk applications where disparities are most pronounced. Furthermore, the literature from LMICs emphasizes the need for tailored implementation approaches. In Africa, for example, ongoing discussions have identified key priorities such as strengthening electricity and internet infrastructure [33], expanding the data science workforce through increased educational opportunities [33], leveraging smartphone-based AI apps [33], and developing AI frameworks that reflect regional needs [34]. In addition, fostering multisectoral partnerships and policy initiatives has been recommended as a way to incentivize and support responsible AI adoption [35].

The trade-offs between transparency, equity, and operational feasibility must also be carefully managed. While transparency fosters trust and accountability, excessive documentation requirements or mandatory external reviews may slow the deployment of ML-driven health interventions, particularly in rapidly evolving crises such as infectious disease outbreaks. In such cases, prioritizing community engagement, establishing advisory panels with representatives from populations considered marginalized, and implementing practical bias mitigation strategies such as integrating socioeconomic variables into model adjustments can ensure ethical safeguards while maintaining efficiency. Flexible regulatory frameworks that accommodate local resource constraints can allow LMICs to adopt ML responsibly without overwhelming already burdened health care infrastructures.

Partnering directly with communities can help address these gaps. This includes building trust, capacity building, and advancing representation [32]. Bias in ML models can stem from various sources, including data collection, label selection, and feature inclusion. Stakeholders from communities considered disadvantaged should be engaged not only to address data biases but also to guide decisions on selecting fair labels and features. For example, using health care expenditure as a proxy for need can introduce bias if spending patterns differ across communities due to economic disparities. Direct involvement of community members can also help identify potential biases in data sources and variables, as well as develop more equitable models. For instance, in the development of predictive models for health care access, community insights can reveal overlooked barriers, such as transport challenges or financial constraints. To mitigate this, ML users should prioritize diverse and representative datasets, monitor error rates and performance levels across different patient groups, and consider the downstream implications (eg, potential impacts on health care access and quality of public health interventions) [32].

True partnership goes beyond consultation; it requires ongoing collaboration and investment in local expertise. Many LMICs and resource-constrained settings lack appropriate safeguards, technical expertise, and infrastructure to implement ML tools effectively. Addressing these gaps requires more than just ethical guidelines. It demands direct investment in skills, resources, and institutional support. Practical strategies to foster capacity building include mentorship programs that connect ML experts in high-income countries with local researchers and policymakers to support training and facilitate knowledge exchange. For example, codeveloping open-access educational resources tailored to public health professionals can help increase AI literacy. Sharing technical resources, such as code and cloud-based computing capacity, can support implementation, provided these efforts are led by LMIC stakeholders. Additional strategies include investing in affordable and scalable ML tools designed for LMICs to ensure usability even with limited infrastructure and fostering partnerships between public health institutions, universities, and community organizations to bridge knowledge gaps. By prioritizing these efforts, ML tools can be developed not only with but also by the communities they aim to serve.

Ultimately, capacity building is a long-term commitment that requires sustained funding, institutional support, and local leadership. While external collaborations provide valuable expertise, true sustainability depends on empowering local communities to take ownership of ML initiatives. This ensures that ML applications in public health are not only effective and transparent but also grounded in the realities and needs of the populations they serve [36].

### Recommendation 2: Use ML in Public Health Emergencies and Other Dynamic, Fast-Paced Situations by Collecting, Analyzing, and Using Population-Wide Deidentified Data

ML can be useful in public health emergencies and other dynamic, fast-paced situations by collecting, analyzing, and using population-wide deidentified data. This process of information gathering and manipulation of deidentified data can be carried out without consent, provided that ethical safeguards are in place and risks related to privacy, misuse, and transparency are actively mitigated.

ML plays a crucial role in gathering, reporting, and analyzing information rapidly, which is vital for mitigating further health damage in dynamic, fast-paced situations such as public health emergencies. According to the United Nations’ Sendai framework, reducing disaster risk involves understanding situational risk, strengthening governance, improving preparedness for an effective response, and allocating resources toward measures that can enhance resilience [37]. Prediction models and protocols, such as evacuation planning, have been used to alleviate the adverse effects of such emergencies. ML has greatly enhanced the ability to monitor information and make timely decisions during emergencies. This has enabled disease outbreak prediction, improved evacuation planning, and optimized the distribution of resources to areas in need [27].

During a public health emergency, the rapid collection of deidentified data without consent may be necessary to ensure timely responses. However, this urgency must be balanced with the potential risks of data misuse, privacy breaches, and lack of accountability, particularly in regions with weaker institutional safeguards. In LMICs and other resource-constrained settings, insufficient regulatory oversight may increase the risk of unauthorized access, data exploitation, or reidentification of individuals.

To minimize these risks while maintaining the efficiency of ML-driven public health responses, organizations and governments should adopt structured ethical frameworks that ensure transparency, accountability, and proportionality in data use. Principles such as FAIR (Findable, Accessible, Interoperable, and Reusable) and CARE (Collective Benefit, Authority to Control, Responsibility, and Ethics) offer structured guidelines for ethical data collection and governance and thus can be used in contexts where regulatory oversight is limited. The FAIR principles focus on making health data widely accessible and reusable, which is essential for rapid public health responses while maintaining data integrity and security. The CARE principles, on the other hand, ensure that data collection respects collective benefit and community authority, particularly in Indigenous communities and those considered marginalized, preventing exploitative data use [38]. In addition to regulatory measures, capacity-building efforts should prioritize the establishment of independent data governance bodies within LMICs to oversee ethical ML implementation. Encouraging multistakeholder collaborations, including engagement with civil society organizations and the communities affected, can further enhance accountability. By embedding these safeguards, public health responses can remain rapid and effective without compromising ethical considerations, even in regions with limited regulatory infrastructure.

Similarly, determining whether individuals or populations would be willing to disclose certain information may be complex. Most people and organizations recognize that some degree of data collection without consultation may be necessary. During the COVID-19 pandemic, researchers effectively used deidentified census data to identify high-risk neighborhoods without individual consent [39]. This approach can serve as a model for using public health data during emergencies while adhering to ethical standards. Ethical frameworks related to consent, privacy of information, and their implications for population and public health, as well as crisis management, can help evaluate the ethical feasibility of proposed solutions, even if they do not address all aspects of the current situation. In their development of an ethical data access framework, Lusignan et al [40] outline that structuring collective as well as organizational thinking and decision-making regarding what is and is not appropriate within a crisis early on makes for improved trust between the data providers and recipients, as expectations may be collaboratively set, negotiated, and ideally fulfilled or exceeded. To accelerate the use of deidentified data in public health responses, ethical frameworks should allow faster-access pathways for researchers who can demonstrate secure data handling and infrastructure. This could involve streamlined approvals for projects with proven privacy measures.

Ideally, this process should occur during the interpandemic phase, using inclusive structures and governance models with oversight powers. This process should determine whether the benefits to society, such as reducing long-term loss of life and health, outweigh the trade-offs involving public interest, privacy breaches, and ethical considerations. To ensure the enforcement of these guidelines in real-world scenarios, particularly where regulatory oversight is lacking, we propose several mechanisms. One approach is to integrate these guidelines into existing public health governance structures, ensuring alignment with established ethical and legal frameworks [41]. In addition, public health institutions and regulatory bodies could establish independent review panels to assess compliance with these ML guidelines [42]. Another enforcement mechanism could be the development of accreditation or certification programs for ML applications in public health, which would require adherence to ethical and transparency standards [43].

### Recommendation 3: Ensure Fairness Across Populations and Mitigate Bias

The level of bias mitigation should be proportional to the context and potential impact of the model, ensuring that even low-risk applications maintain essential fairness safeguards. All aspects of the model’s implementation should be assessed, including inherent, external, or incompletely known factors that could contribute to bias.

Risk assessment is central to any innovation, but it is especially important for ML, which is prone to certain liabilities [44]. The use of algorithms in health care can perpetuate racial biases due to existing disparities in health care delivery. When designing algorithms to predict health outcomes based on genetic findings, bias may arise if there is limited or no research conducted in certain populations. For instance, using data from the Framingham Heart Study to predict cardiovascular risk in non-White populations has led to biased results, overestimating or underestimating the risk [45].

To clarify how risk relates to bias mitigation, risk should be defined based on the potential for harm or inequity, particularly to populations considered vulnerable, rather than solely on model complexity or scope. Risk assessment should consider the model’s intended use, the sensitivity of the outcome (eg, health care access or resource allocation), and the potential for systemic harm if biases are present. Importantly, a lower risk designation should never justify a lax approach to bias mitigation. Instead, risk levels should guide the *type* of mitigation strategies deployed, with simpler models undergoing appropriate fairness checks and more complex, high-impact models requiring comprehensive audits and subgroup analyses.

Given the wide range of ML models, conducting situation-specific risk assessments, especially for populations that may be vulnerable due to social or economic policies, is essential. We need to develop protocols to ensure fairness across diverse populations, using metrics such as subgroup performance, false-positive or false-negative rates, and bias-specific checks (eg, checking for overrepresentation or underrepresentation of particular groups). Transparency should extend to data sources, model methodologies, and decisions made during development. Providing accessible documentation, including technical reports and simplified explanations, can help both technical and nontechnical stakeholders understand potential biases and their mitigation [32]. Transparency should highlight how different populations are represented in training data and how model outputs vary among them. For example, models predicting health care access should clearly show performance disparities between urban and rural populations.

Transparency, explainability, and interpretability of ML models are critical for stakeholder trust and decision-making. Transparency refers to how openly the model’s design, data sources, and decision-making processes are shared, helping stakeholders understand how the model operates. Efforts should be made to improve the transparency of models, such as through clear explanations of how inputs influence outputs, visual aids, and plain language summaries. Explainability focuses on making specific predictions understandable, clarifying why a model produced a particular output through methods such as feature importance analysis or decision trees. Interpretability relates to how easily a human can understand the model’s internal logic or decision-making process, which is often linked to model simplicity or the use of interpretable techniques such as linear regression or rule-based systems. Altogether, these concepts ensure that ML models are accessible, trustworthy, and accountable to both technical and nontechnical stakeholders.

For example, using interpretable models in clinical settings can help physicians understand and validate AI recommendations, enhancing adoption and reliability. ML is sometimes portrayed as a technology requiring advanced training to understand, leading it to both hopeful expectations and suspicion. It is important to clearly communicate the inherent and associated risks of the model and define what is meant by ML in its context [32]. When ML-related technologies and their risks are explained in plain language, public distrust tends to decrease [46].

### Recommendation 4: Ensure Public Availability of Data Sources, Model Methodologies, and Technical Details, Along With Bias Mitigation Strategies, Used in the Contexts of Models to Promote Transparency, Reproducibility, and Trustworthiness Across ML Studies

Providing accessible information about the technical aspects of an ML study or solution, such as its data sources, population, characteristics, and model variables, along with detailed descriptions of its methodology and the deidentified datasets used, supports reproducibility, bias mitigation, and trustworthiness [32]. However, ensuring public availability of data must be balanced with the sensitivity of health data, privacy regulations, and proprietary constraints. To promote meaningful transparency, a structured approach is necessary, one that goes beyond simply sharing code and considers regulatory, ethical, and contextual limitations. Drawing on European Union legislation and literature in computer science, Kiselva and De Hert [47] suggest that transparency must be seen as a fundamental “way of thinking” and an all-encompassing concept that characterizes the process of developing and using AI.

Achieving transparency requires balancing openness with the risks of reidentification, legal restrictions, and proprietary interests. While transparency enhances trust in ML models, full public disclosure of health data is not always feasible due to privacy laws such as the Health Insurance Portability and Accountability Act (HIPAA) and General Data Protection Regulation (GDPR), institutional data-sharing policies, and concerns over commercial confidentiality.

In public health contexts, transparency should function as a system of accountability rather than unrestricted access to all datasets. ML documentation should be structured to ensure usability while maintaining compliance with privacy and proprietary safeguards. This includes providing methodological details and bias mitigation strategies, ensuring that plain language summaries are available for nontechnical stakeholders, and adopting tiered-access models where sensitive data can be reviewed under controlled conditions rather than being made fully public. The need for transparency should also be weighed against the practical barriers to data sharing. Health data often originate from multiple institutions with varying governance policies, making harmonization difficult. In addition, proprietary models developed by industry partners may involve intellectual property protections that restrict full disclosure of methodologies. Addressing these challenges requires the development of standardized documentation and data governance agreements that balance the need for public trust with confidentiality concerns.

To enhance data sharing, transparency, and bias mitigation, dataset development protocols should align with existing frameworks to integrate new and prior information. Data used for model training, validation, or implementation should be of high quality (eg, completeness, source consistency, and linkage potential) and consistently accessible and updated, especially data related to employment, education, occupation, other socioeconomic status factors, and health inequalities [48]. In applications of linked data and ML in the health sciences (eg, to estimate population-level health indicators), bias can arise from nonstandard data collection methodologies. Developing standardized protocols for all data sources used in each project and including variance assessment and context-appropriate handling (eg, oversampling, imputation) in bias mitigation strategies are essential. For example, in response to bias concerns during the COVID-19 pandemic, ML models were adjusted to incorporate sociodemographic data to improve accuracy in predicting infection spread among populations considered marginalized [49]. Another successful example includes the use of algorithmic audits in health care AI applications, where independent researchers identified and mitigated racial biases in predictive models used for hospital readmission rates [50]. Transparency should include public documentation of these bias mitigation strategies while ensuring that sensitive details remain protected.

Assessing bias and performance across subpopulations is another essential element of responsible transparency. For public health surveillance, sensitivity may be prioritized to avoid missing cases, whereas models used for treatment decisions may emphasize specificity to minimize unnecessary interventions. Metrics such as area under the curve, positive predictive value, and user satisfaction should be evaluated across different subpopulations to detect and correct bias. It is equally important to identify variance in datasets, as high variance can exacerbate bias in ML models. Underfitting, which occurs when a model fails to capture important patterns, and overfitting, where a model becomes hypersensitive to minor fluctuations in data, should be addressed through continuous model tuning and hyperparameter optimization.

In addition to predeployment fairness checks, postdeployment monitoring should include regular performance evaluations across diverse subpopulations to detect bias drift, which can emerge as population characteristics change. Bias drift occurs when the model’s predictive performance deteriorates or produces skewed results over time, often reflecting shifts in population demographics or health care access patterns. We also highlight the importance of model update protocols incorporating community feedback loops, participatory audits, and transparent reporting of adjustments to ensure that updates are responsive and equitable.

We recommend that organizations implement structured, iterative audit cycles to support these processes. These cycles should include predeployment fairness checks using simulated population data and postdeployment ethical audits conducted at regular intervals or following significant model updates. In addition, establishing rapid-response mechanisms to address emerging ethical concerns during deployment can help maintain accountability. Incorporating stakeholder feedback from impacted communities through participatory reviews ensures that evaluations remain inclusive and socially responsive.

### Recommendation 5: Facilitate Regular, Multidisciplinary Discussions Involving ML Developers, Public Health Professionals, Ethicists, and Community Representatives to Identify Biases, Ensure Fair Implementation, and Increase Transparency

Provide plain language summaries and guidelines that are consistent in terminology to raise awareness among both the public and experts about ML-related bias and debiasing.

Efforts to raise public awareness about ML and its benefits should be balanced with accurate information about potential harms that may be associated with its implementation. In a study by Musbahi et al [51], the views of patients and the public about AI in health care were analyzed. The top 5 concerns included decreased human interaction, data security, obtaining consent for data use, errors in AI systems, and the potential irrelevance of AI in health care. Despite factors promoting the adoption of technology in health care settings, achieving sustainable implementation remains a challenge. Providing information to address individual concerns about the safety and effectiveness of ML models is essential. Public disclosure and scientific interrogation of potentially harmful occurrences or risks associated with ML, including unintentional biases, build trust with the public. Efforts to raise awareness should use accessible language and incorporate research best practices (eg, consistent terminology, structured abstracts, and established reporting guidelines) to make technical concepts easier to understand for a wider audience [46]. This approach not only raises awareness but also fosters trust among nontechnical stakeholders, such as community members and health care practitioners. Past multidisciplinary engagements have proven valuable in addressing bias and transparency concerns in ML applications. For instance, the World Health Organization convened multistakeholder panels to assess AI-based disease surveillance tools, leading to refined ethical guidelines [52]. Similarly, the Canadian AI for Public Health initiative facilitated workshops bringing together data scientists, epidemiologists, and policymakers to discuss bias mitigation strategies in ML-based public health interventions [53]. A structured approach to future engagements could include regular stakeholder summits, interdisciplinary task forces, and community-centered consultations.

## Summary

ML is rapidly advancing and holds potential for uses to improve the health of individuals and communities. However, these efforts must prioritize equity. Model developers, statisticians, epidemiologists, public health professionals, policymakers, and funders must collaborate to ensure that ML implementations avoid prejudice and discrimination while also enhancing human capabilities, connections, and knowledge in health and disease contexts. This approach aligns with the ethical integration of AI in health [54].

Our recommendations align with existing frameworks. In the United States, our guidelines support the principles outlined in the Executive Order on Safe, Secure, and Trustworthy AI, which emphasizes the importance of equity, privacy safeguards, and risk mitigation in health-related AI applications [55]. Similarly, our focus on transparency and bias mitigation aligns with the European Union’s AI Act and GDPR, which collectively promote ethical AI deployment, data protection, and algorithmic accountability [56,57]. Our recommendations complement the UK AI Regulatory Principles, which emphasize fairness, accountability, and transparency, particularly in public sector applications [58]. Finally, our guidelines build upon principles from the Pan-Canadian Artificial Intelligence Strategy [59], which highlights equity, interdisciplinarity, and inclusivity as core pillars of responsible AI adoption in public health. These country-specific frameworks emphasize the importance of adapting ethical ML guidelines to regional regulatory and social contexts, while ensuring global alignment on core principles such as equity, transparency, and data privacy. Our recommendations add to these discussions by offering a public health–specific lens, focusing on bias mitigation in population-level models, fostering community partnerships, and emphasizing the impact of social determinants of health in ML implementation.

We had a diverse range of disciplines and approaches represented in our team, including AI, ML, computer science, population health, epidemiology, ethics, and AI applications in health. A limitation of this work was that we have adapted methodologies initially designed for developing clinical practice guidelines, as there is no clear guidance for developing ML applications in population and public health. In LMICs, there might be limited resources available for adequately monitoring and documenting public health interventions that use ML. While our recommendations aim to promote equity and enhance the integration of ML models in LMICs for population and public health purposes, they may not cover all possible use cases. Implementing these guidelines in diverse settings presents several challenges, including variations in data quality, resource availability, and stakeholder engagement. Addressing these challenges is essential to ensure that ML models enhance public health outcomes equitably.

## Limitations

Some key limitations include difficulties in engaging communities considered disadvantaged, ensuring data representativeness, and maintaining transparency with limited resources. Operationalizing these recommendations will require adaptable protocols, increased local capacity, and collaborative efforts. For example, developing simplified, context-specific inclusivity checklists and leveraging international partnerships can help overcome resource gaps. Ultimately, ongoing evaluation and stakeholder input are crucial for refining these guidelines and ensuring their effectiveness across varied public health contexts.

## Conclusions

Without adequate bias prevention and mitigation during ML model development and implementation, ML applications in population and public health contexts could worsen existing health disparities or even contribute to new ones. Similar to the development of health policy aimed at promoting equity, it is crucial to carefully assess ML innovations before, during, and after their development and deployment. This ensures that model design and delivery are equitable and based on data representing all groups. Achieving equity requires adhering to ethical principles of equity, transparency, and engagement, as well as multidisciplinary efforts involving both model developers and population health practitioners committed to bias prevention and mitigation standards. Monitoring the outcomes of such adherence, including the guidelines proposed here, can promote less biased use of ML to inform policy. Future work should include piloting these guidelines in a range of public health settings, including LMICs, and developing practical tools to assess their effectiveness. Evaluation efforts could involve tracking the diversity of stakeholders involved in model development and testing, measuring improvements in model accuracy for underrepresented groups, and assessing whether decisions based on these models contribute to more equitable health outcomes. Ongoing assessment and adaptation of these guidelines will be crucial for ensuring responsible and fair use of ML in public health. ML is not just a rapidly evolving technology but also a tool that can promote equity in population and public health if a commitment to mitigating bias is maintained by all stakeholders involved in its design and delivery.

## Supplementary material

10.2196/68952 Multimedia Appendix 1 Guideline team composition.

## Abbreviations

AI: artificial intelligence
CARE: Collective Benefit, Authority to Control, Responsibility, and Ethics
FAIR: Findable, Accessible, Interoperable, and Reusable
GDPR: General Data Protection Regulation
GRADE: Grading of Recommendations Assessment, Development and Evaluation
HIPAA: Health Insurance Portability and Accountability Act
LMIC: low- and middle-income country
ML: machine learning
NCD: noncommunicable disease
NICE: National Institute for Health and Care Excellence
