# Supplementary material for: Machine Learning Applications in Population and Public Health: Guidelines for Development, Testing, and Implementation
Source: JMIR Public Health Surveill. 2025 Oct 24;11:e68952. doi: 10.2196/68952 (PMC12551935; doi:10.2196/68952)
Supplement: Multimedia Appendix 1 [file publichealth-v11-e68952-s001.docx]

**Appendix 1: Guideline team composition**

| **Andrew Pinto**  Public health specialist and clinician-scientist, director of the Upstream Lab; Associate Professor, University of Toronto. Expertise in integrating social data into EMRs, AI applications in primary care. AI lead for the Dept. of Family and Community Medicine, University of Toronto. |
| --- |
|  |
| **Sharon Birdi**  Research Coordinator, Upstream Lab, MAP Centre for Urban Health Solutions; knowledgeable in health services research, social determinants of health, and critical social research. |
|  |
| **Steve Durant**  Research Coordinator, Upstream Lab, MAP Centre for Urban Health Solutions; expertise in health services research, social determinants of health, and critical social research. |
|  |
| **Shehzad Ali**  Canada Research Chair in Public Health Economics at the Department of Epidemiology and Biostatistics, Schulich School of Medicine and Dentistry. Expertise in health services performance evaluation in terms of efficiency and equity of access, utilization and patient outcomes; Developing statistical algorithms for predicting patient outcomes in clinical practice. |
|  |
| **David Buckeridge**  Public Health Informatics specialist, and Professor in the Department of Epidemiology, Biostatistics and Occupational Health at McGill University. Expertise in automated surveillance to guide public health interventions. |
|  |
| **Marzyeh Ghassemi**  Computer scientist, AI specialist, visiting researcher at Verily/Google. Expertise applying various machine learning methods to improve health care. |
|  |
| **Jennifer Gibson**  Director of the Joint Centre for Bioethics, Chair in Bioethics at the University of Toronto. Leads the “Ethics and AI for Good Health” program. |
|  |
| **Ava John-Baptiste**  Associate Professor, Department of Epidemiology and Biostatistics, Interfaculty Program in Public Health, Western University. Expertise in use of models to inform policy and adequacy of COVID-19 models for protection of socially and occupationally vulnerable populations. |
|  |
| **Melissa McCradden**  PhD bioethicist at The Hospital for Sick Children and a member of the CONSORT-AI and SPIRIT-AI Working Group establishing the mandatory reporting standards for clinical trials involving AI interventions. Her work focuses on algorithmic fairness solutions, paediatric bioethics, and explainability in a shared decision-making environment. |
|  |
| **Kwame McKenzie**  CEO of the Wellesley Institute and Director of Health Equity at the Centre of Addiction and Mental Health (CAMH). Expertise in social causes of illness and health equity. |
|  |
| **Sharmistha Mishra**  Canada Research Chair in Mathematical Modeling and Program Science. Expertise in model comparison, with a particular focus on biases generated by inclusion/exclusion of causal pathways of disease transmission in predictive and intervention modelling. |
|  |
| **Akwasi Owusu-Bempah**  Assistant Professor in the Department of Sociology UofT and affiliate scientist, CAMH. Expertise in the intersection of race, crime, policing, justice, and social inequality. |
|  |
| **Laura Rosella**  Associate Professor at University of Toronto in public health, appointed to Public Health Ontario, ICES and Vector Institute. Expertise in predictive models that use large datasets. |
|  |
| **James Shaw**  Scientist at the Women’s College Hospital Institute, and Research Director of Artificial Intelligence (AI), Ethics & Health at the University of Toronto Joint Centre for Bioethics. Expertise in implementation and ethical implications of innovations in health care with a special focus on digital health technologies and applications of AI in health care. |
|  |
| **Ross Upshur**  Professor and Head of the Division of Clinical Public Health in the Dalla Lana School of Public Health and Department of Family and Community Medicine, University of Toronto, Senior Scientist, Lunenfeld Tanenbaum Research Institute Sinai Health, Toronto. Experienced in research intersecting primary care and public health, particularly with respect to the interrelationships between ethics and evidence. Experienced in research intersecting primary care and public health, particularly with respect to the interrelationships between ethics and evidence. |
|  |
| **Parisa Naraei**  Computer scientist and manager, CIHI; expertise in applied deep learning and machine learning algorithms to solve various problems in the healthcare domain. |
|  |
| **Jillian Macklin**  MD/PhD(c) at U of T with focus on Health Services research at IHPME. Expertise in ethical concerns of AI applications and health, and designing patient engagement frameworks and activities for meaning participation of research. |
